# *Cinnamomum cassia* Bark in Two Herbal Formulas Increases Life Span in *Caenorhabditis elegans* via Insulin Signaling and Stress Response Pathways

**DOI:** 10.1371/journal.pone.0009339

**Published:** 2010-02-22

**Authors:** Young-Beob Yu, Laura Dosanjh, Lixing Lao, Ming Tan, Bum Sang Shim, Yuan Luo

**Affiliations:** 1 Department of Pharmaceutical Sciences, School of Pharmacy, University of Maryland, Baltimore, Maryland, United States of America; 2 Center for Integrative Medicine, School of Medicine, University of Maryland, Baltimore, Maryland, United States of America; 3 Division of Biostatistics, School of Medicine, University of Maryland, Baltimore, Maryland, United States of America; 4 Brain Disease Research Center, Korea Institute of Oriental Medicine, Daejeon, Korea; 5 Department of Medicinal Plant Resources, Nambu University, Gwangju, Korea; 6 College of Oriental Medicine, Kyunghee University, Seoul, Korea; Purdue University, United States of America

## Abstract

**Background:**

Proving the efficacy and corresponding mode of action of herbal supplements is a difficult challenge for evidence-based herbal therapy. A major hurdle is the complexity of herbal preparations, many of which combine multiple herbs, particularly when the combination is assumed to be vitally important to the effectiveness of the herbal therapy. This issue may be addressed through the use of contemporary methodology and validated animal models.

**Methods and Principal Findings:**

In this study, two commonly used traditional herbal formulas, *Shi Quan Da Bu Tang* (SQDB) and *Huo Luo Xiao Ling Dan* (HLXL) were evaluated using a survival assay and oxidative stress biomarkers in a well-established *C. elegans* model of aging. HLXL is an eleven herb formula modified from a top-selling traditional herbal formula for the treatment of arthritic joint pain. SQDB consists of ten herbs often used for fatigue and energy, particularly in the aged. We demonstrate here that SQDB significantly extend life span in a *C. elegans* model of aging. Among all individual herbs tested, two herbs *Cinnamomum cassia* bark (Chinese pharmaceutical name: Cinnamomi Cortex, CIN) and *Panax ginseng* root (Chinese pharmaceutical name: Ginseng Radix, GS) significantly extended life span in *C. elegans*. CIN in both SQDB and HLXL formula extended life span via modulation of multiple longevity assurance genes, including genes involved in insulin signaling and stress response pathways. All the life-span-extending herbs (SQDB, CIN and GS) also attenuated levels of H_2_O_2_ and enhanced small heat shock protein expression. Furthermore, the life span-extending herbs significantly delayed human amyloid beta (Aβ)-induced toxicity in transgenic *C. elegans* expressing human Aβ.

**Conclusion/Significance:**

These results validate an invertebrate model for rapid, systematic evaluation of commonly used Chinese herbal formulations and may provide insight for designing future evidence-based herbal therapy(s).

## Introduction

Use of botanic supplements has increased substantially in the last decade in the United States. The efficacy and mechanisms of herbal supplements are largely unknown. A consumer survey in the U.S. determined that 49% of all American adults had used at least one herbal medicine during the previous year [1], with 24% acknowledging that they used herbs on a regular basis [2]. The disease prevention theory associated with natural medicine has the potential to both increase quality of life and reduce health care costs in the United States [3]. The proof of efficacy of herbal preparations and determination of their mode of action are difficult challenges for evidence-based herbal therapy. As the National Center for Complimentary and Alternative Medicine (NCCAM) program developed, it became clear that high-quality basic and pre-clinical research is a necessary precursor to clinical studies and should employ contemporary research technologies to decode the complexity of botanicals.

Botanical research has focused for decades on the search for a single most active ingredient in a plant, based on the assumption that the plant has one or more ingredients which determine its therapeutic effect. However, traditional Chinese medicine (TCM) and European phytomedicine generally assert that synergy is vitally important for the effectiveness of herbal therapy. Herbal medicine has existed for more than 5000 years. Today there are more than 3000 kinds of medicinal herbs. Although each herb has individual indications, in classical Chinese herbal medicinal practice diseases are commonly treated by combining herbs into formulas. In the early Ming dynasty (1368–1644 A.D.), more than 60,000 herbal prescriptions/formulas were recorded. These herbal combinations are believed to act synergistically to harmonize beneficial effects and to neutralize or minimize the toxic or adverse effects of individual constituent herbs. However, this claim has not been scientifically evaluated.

In recent years, numerous efforts have been made, particularly in China and other Asian countries, to investigate the mechanisms of action of Chinese herbal formulas using modern scientific methodology [4]–[6]. *In vitro* and *in vivo* studies on the individual herbs or constituents of classic formulas have been reported that many herbal medicines that are components of widely recognized traditional Chinese herbal formulas have immunomodulatory or antioxidant effects that may offer clinically relevant therapeutics for patients with disorders associated with aging [7], [8]. While the majority of research has focused on the identification of the biological functions and active components of individual constituents, there are relatively few reported studies that consider the effects of interaction among herbs within a formula.

Working with highly complex multicomponent formulas with multiple possible interactions between component herbs requires studies in multicellular organisms. Because the systematic evaluation of efficacy, safety and mechanism of these multi-herbal formulas in mammalian models is costly and time consuming, we chose the well established nematode *Caenorhabditis elegans*, a valuable model for studies of aging and age-related disorders [9], [10], [11], and an attractive platform for rapidly screening drug safety and efficacy [12]. Using this model we have previously demonstrated life span prolonging and neuroprotective mechanisms of *Ginkgo biloba* extract EGb 761 [13]–[18], protection against sarcopenia by ginseng [19] and delays of age-related functional declines by green tea extract EGCG [20].

Genetic and pharmacological modification of lifespan mechanisms has been well elucidated in *C. elegans*, fly and mice [21]. Although translation of life span extension from worm to humans is unknown, substantial experimental evidence suggest that life-span extending drugs are extremely useful in treatment of age-related diseases such as cancer and neurodegenerative diseases [22]–[24]. The present study employs a systematic biological approach to reveal herbal formula mechanism of action in an invertebrate *C. elegans* model that shares high homology with mammalian and human genes and biochemical pathways [10].

HLXL is an herbal formula modified from a top-selling traditional herbal formula for management of chronic inflammatory pain [25]. No side effects were listed at clinical doses of the 11 individual herbs in the formulation of HLXL (Table 1). Our previous studies have demonstrated that HLXL significantly inhibited inflammation and hyperalgesia in CFA-induced arthritis in rat [26], [27]. Shi-Quan-Da-Bu-Tang SQDB (Ten Significant Tonic Decoction) or Sipjeondaebotang, Juzentaihoto, (TJ-48) consists of 10 component herbs (Table 1). SQDB has been widely reported as a treatment for a variety of conditions including aging related disorders, though it is primarily used to enhance quality of life and immune function [28], [29]. Recently, SQDB was found to induce intestinal microflora which, in turn, altered HSP gene expression [30]. Age-related declines of brain monoamines in mice were restored to levels observed in young mice by SQDB treatment [31]. SQDB also exhibits immunomodulatory [32], [33], anti-angiogenic, and anti-tumor effects [34], [35]. A clinical trial conducted in Japan demonstrated that patients in a treatment group that consumed 7.5 g as a daily oral dose of SQDB for up to six years had significantly longer cancer remission survival time (49 months) as compared to the control group (24 months, p = 0.023) and no adverse effects were observed during the six years of treatment [36].

**Table 1:** Botanical names and origin of the formulas SQDB and HLXL.

SQDB	HLXL
*Systematic name* [plant part] [Pharmaceutical name, Chinese name]	*Systematic name* [plant part] [Pharmaceutical name, Chinese name]
*Angelica gigas* [root] [Angelicae Gigantis Radix, Danggui]	*Angelica sinensis* [root] [Angelicae Sinensis Radix, Danggui]
*Astragalus membranaceus* [root] [Astragali Radix, Huangqi]	*Angelica pubescens* [root] [Angelicae Pubescentis Radix, Duhuo]
*Atractylodes japonica* [rhizome, white] [Atractylodis Rhizoma Alba, Baizhu]	*Boswellia carterii* [resin] [Olibanum, Ruxiang]
*Cinnamomum cassia* [bark] [Cinnamomi Cortex, Guizhi]	*Cinnamomum cassia* [bark] [Cinnamomi Cortex, Guizhi]
*Cnidium officinale* [rhizome] [Cnidii Rhizoma, Chuanxiong]	*Lingustium chuanxiong* [rhizome] [Chuanxiong Rhizoma, Chuanxiong]
*Glycyrrhiza uralensis* [root] [Glycyrrhizae Radix, Gancao]	*Glycyrrhiza uralensis* [root] [Glycyrrhizae Radix, Gancao]
*Paeonia lactiflora* [root] [Paeoniae Radix, Chishao]	*Paeonia lactiflora* [root] [Paeoniae Radix, Chishao]
*Panax ginseng* [root] [Ginseng Radix, Renshen]	*Corydalis yanhusuo* [rhizome] [Corydalis Rhizoma, Yanhusuo]
*Poria cocos* [sclerotium] [Poria Sclerotium, Fuling]	*Gentiana macrophylla* [root] [Gentianae Macrophyllae Radix, Qinjiao]
Rehmannia glutinosa [root] [Rehmanniae Radix Preparata, Shudihuang]	*Notopterygium incisum* [root] [Notopterygii Radix, Qianghuo]
	*Salvia miltiorrhiza* [root] [Salviae Miltiorrhizae Radix, Danshen]

In this study, we seek to test the hypothesis that traditional herbal formulas protecting against age-related disorders in humans can be evaluated in an animal model of aging using assays of biomarkers and age-associated functional changes shared between nematodes and humans.

## Results

### 1. Herbs *Cinnamomum cassia* Bark (CIN), *Panax ginseng* Root (GS), and Formula SQDB Significantly Prolong Life Span in *C. elegans*

To determine whether the two commonly used herbal formulas extend life span in *C. elegans*, wild type *C. elegans* (N2, Table 2) were grown in liquid media containing either vehicle or crude herbal formula SQDB, HLXL, or individual herbs, using a high throughout liquid culture assay. Adult wild type worms grown under these conditions at 20°C have a mean life span of 19.9 days. In *C. elegans* fed with SQDB (100 µg/ml in food), mean life span of the worms was significantly prolonged by 11.7% (mean life span 22.3 days, n = 3 independent experiments, total 555 worms, p = 0.0004, Table 3). As a positive control, mianserin, which was previously shown to extend life span in *C. elegans* under similar conditions [22] also significantly extended mean life span by 35.9% (mean life span 27.1 days, n = 4 experiments, p = 0.00001, total 637 worms). In contrast, formula HLXL (100 µg/ml) did not extend mean life span significantly (mean life span 20.7 days, p = 0.1364, total worms 139, Table 4).

**Table 2:** Description of *C. elegans* strains.

Strain	Gene	Expression	Phenotype	Assay
N2	Wild type			Life span
*ser-1*(ok345)	5-HT receptor		Increased life span	Life span
*daf-16* (mgDf50)	Insulin pathway		Decreased life span	Life span
*mev-1*(kn1)III	Electron transport		ROS sensitive	Life span
CL4176	*myo-3*/Aβ 42	Inducible muscle Aβ	Rapid paralysis	Paralysis
CL2070	GFP reporter for sHSPs	GFP		Thermal, oxidative stress

**Table 3:** Life span assays in wild type *C. elegans* fed with SQDB and individual herbs.

N2 strain	No. of experiments	Mean life span (days)	% change	No. of animal (+/−herbs)	p-value
Control	4	19.9		322	
Mianserin	4	27.1	35.9	315/322	0.0000
SQDB	3	22.3	11.7	233/322	*0.0004
*Angelica gigas*	2	20.0	0.2	65/322	n.s. (0.8317)
*Astragalus membranaceus*	2	20.9	4.8	70/322	n.s. (0.5422)
*Atractylodes japonica*	2	19.7	−1.1	53/322	n.s. (0.6144)
*Cinnamomum cassia* (CIN)	3	22.1	10.8	170/322	*0.0017
*Cnidium officinale*	2	19.8	−0.8	56/322	n.s. (0.9064)
*Glycyrrhiza uralensis*	2	20.7	3.6	62/322	n.s. (0.9328)
*Paeonia lactiflora*	2	19.4	−2.8	58/322	n.s. (0.2946)
*Panax ginseng* (GS)	3	21.5	7.7	167/322	*0.047
*Poria cocos*	2	19.8	−0.9	72/322	n.s. (0.3162)
*Rehmannia glutinosa*	2	20.9	4.7	60/322	n.s. (0.9481)

*P<0.005, Statistical significant; n.s. P>0.005 not significant.

**Table 4:** Life span assays in wild type *C. elegans* fed with HLXL and individual herbs.

N2 strain	No. of experiments	Mean life span (days)	% change	No. of animals	p-value
Control	3	19.5		141	
HLXL	2	20.7	5.7	139	n.s.(0.1364)
*Angelica pubescens*	1	21.0	7.5	35	n.s.(0.1892)
*Angelica sinensis*	1	19.2	−2.0	39	n.s.(0.5373)
*Lingustium chuanxiong*	1	17.0	−13.0	29	n.s.(0.4937)
*Cinnamomum cassia* (CIN)	2	22.4	14.5	93	*0.0041
*Corydalis yanhusuo*	1	17.7	−9.4	30	n.s. (0.3039)
*Gentiana macrophylla*	1	20.2	3.2	35	n.s.(0.2190)
*Glycyrrhiza uralensis*	1	19.8	1.3	34	n.s.(0.8503)
*Notopterygium incisum*	1	19.2	−1.7	20	n.s.(0.3520)
*Boswellia carterii*	2	20.9	7.1	101	n.s.(0.0744)
*Paeonia lactiflora*	1	19.4	−0.9	39	n.s.(0.8799)
*Salvia miltiorrhiza*	2	21.2	8.3	85	n.s.(0.0539)

*P<0.005, Statistical significant; n.s. P>0.005 not significant.

SQDB and HLXL contain 11 and 10 individual herbs, respectively. To determine which herb(s) contribute to life span prolongation, we assayed each individual herb for effects on *C. elegans* survival. Among all individual herbs tested, only two herbs *Panax ginseng* root (GS) and *Cinnamomum cassia* bark (CIN) significantly extended life span in *C. elegans* (Table 3). *Cinnamomum cassia* bark present in both herbal formulas significantly extended mean lifespan to a similar extent (Table 3 and 4).

### 2. Life Span Extension by *CIN* (*Cinnamomum cassia* Bark) Requires Multiple Cellular Pathways

Cellular pathways for genes that affect life span have been identified in *C. elegans*. Life span extension by CIN offers an opportunity to use the power of worm mutants to test mechanisms of action. This approach was applied in our recent publication [17] showing that inhibition of 5-HT-controlled egg laying by bilobalide is blocked in *mod-1 and ser-4* suggesting a role for 5-HT receptors in the beneficial effects of bilobalide. A similar approach also led to the identification of resveratrol, a natural compound that extends the worm's life span by acting on the *Sir2* gene [37], and the requirement of the CaMKII pathway for osmotic stress resistance and life span extension by blueberry extracts [38].

**Figure 1:**
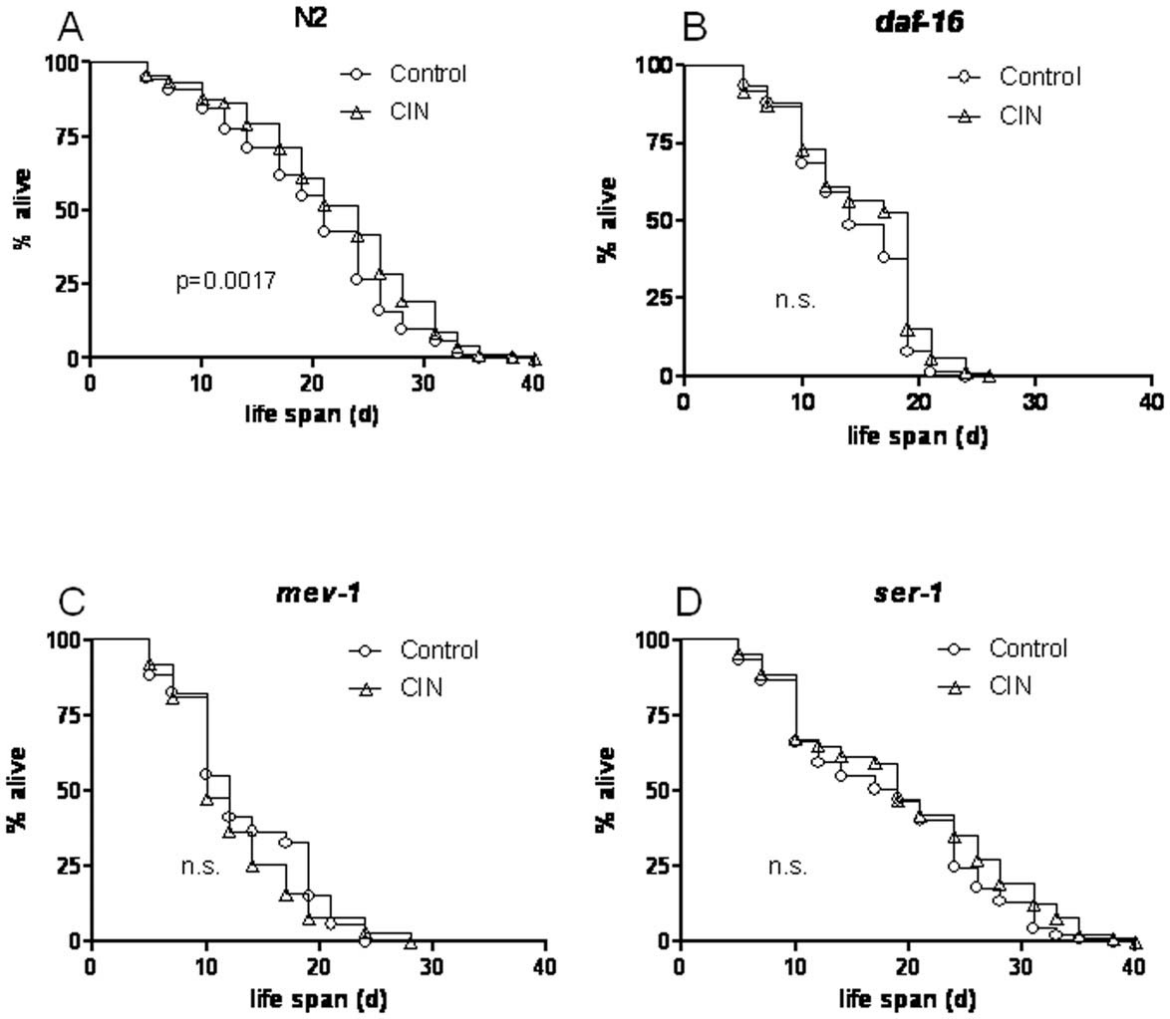
Effect of *Cinnamomum cassia* (CIN) on life span in multiple pathway mutants of *C. elegans*. Age synchronized control (circles) or CIN fed (triangles) worms were seeded in liquid medium at the L1 stage. CIN treatment began 68 h after seeding (day 1 of adult life) and the fraction of animals alive was scored microscopically on the basis of movement. A. CIN significantly extends life span in wild type worms but not in B. *daf-16* C. *mev-1* or D. *ser-1* mutants. Survival rate was scored every day and is expressed as percentage of survival. The experiment was conducted at least three times with a minimum of 100 worms per trial. Statistical significant p<0.05, n.s. not significant.

To identify the mechanism of action of CIN, we examined the effects of CIN on life span in three mutants implicated in major longevity pathways. If a specific pathway were required for lifespan extension by CIN, then CIN would be unable to prolong lifespan in that mutant. In *C. elegans*, the DAF-16 transcription factor in the insulin signaling pathway promotes expression of stress resistance and longevity genes. In mutant worms (*daf-16*) fed with CIN, the ability of CIN to extend lifespan was impaired compared to vehicle fed controls (Fig. 1A&B, Table 5). Similarly, lifespan extension by CIN was also blocked in mutants of serotonin receptors (*ser-1*) and anti-oxidative enzyme (*mev-1*) (Fig. 1C&D, Table 5), suggesting that CIN requires the insulin signaling pathway, anti-oxidative pathway and serotonin signaling for its lifespan prolonging effect. Lifespan extension by ginseng (GS), on the other hand, was unaffected in the *daf-16* mutant (Table 5 & 6) indicating that ginseng may act independently of this gene. Interestingly, the herbal formula SQDB prolonged life span in *ser-1* mutant worms (Table 5 & 6), suggesting that the serotonin pathway may not be involved in lifespan prolongation by SQDB.

**Table 5:** Life span assays in mutant *C. elegans* fed with SQDB and individual herbs.

Strains	Drugs	Mean life span (d)	% Change	No. of animal (+drug/−drug)	P-value
	Control	14		76	
	SQDB	15	7.1	57/76	n.s. (0.295)
	HLXL	15	7.1	64/76	n.s. (0.8088)
*Daf-16*	*Cinnamomum cassia* (CIN)	15	7.1	85/76	n.s. (0.0596)
	*Boswellia carterii*	14	0.0	89/76	n.s. (0.8369)
	*Panax ginseng* (GS)	17	21.4	72/76	*0.0006
	Control	18		89	
	SQDB	22.4	24.4	100/89	*0.0045
	HLXL	17.6	−2.2	100/89	n.s. (0.3203)
*Ser-1*	*Cinnamomum cassia* (CIN)	19.8	10.0	88/89	n.s. (0.1299)
	*Boswellia carterii*	21.3	18.3	57/89	n.s. (0.1054)
	*Panax ginseng* (GS)	19.5	8.3	80/89	n.s. (0.3296)
	Control	13		85	
	SQDB	12	−7.7	56/85	n.s. (0.3368)
	HLXL	13	0.0	66/85	n.s. (0.4368)
*Mev-1*	*Cinnamomum cassia* (CIN)	13	0.0	63/85	n.s. (0.3506)
	*Boswellia carterii*	13	0.0	61/85	n.s. (0.508)
	*Panax ginseng* (GS)	14	7.7	69/85	n.s. (0.416)

*P<0.005, Statistical significant; n.s. P>0.005 not significant.

**Table 6:** Summary.

Assay	Strain		% Change (p value)		
		SQDB	GS	CIN	Statistics
Life span	N2	11.7 (0.0004)	7.7(0.047)	10.8(0.0017)	Log-rank test
	*Ser-1*	24.4(0.0045)	8.3 (0.3296)	10.0(0.1299)	
	*Daf-16*	7.1(0.295)	21.4(0.0006)	7.1(0.0596)	
	*Mev-1*	(−)7.7(0.3368)	7.7(0.416)	0(0.3506)	
ROS	CL-2070	18.5*	14.9*	25**	T-test
Thermo-tolerance	CL-2070	21.6***	18.2**	21.5***	T-test
Paralysis	CL-4176	P<0.0004	P<0.0002	P<0.0164	Log-rank test

### 3. Life Span-Extending Herbs Enhance Expression of Small Heat Shock Proteins (sHSP16) and Attenuate Intracellular Levels of H_2_O_2_ in *C. elegans*

**Figure 2:**
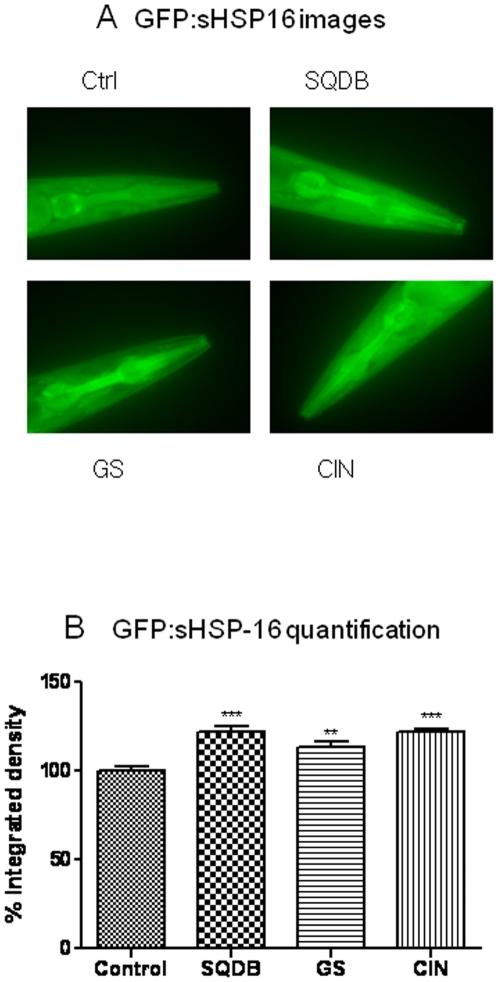
Representative epifluorescence image of transgenic *C. elegans* (CL2070, hsp-16- 2/GFP) fed with or without herbs followed by a heat-shock treatment for 2 hr. CL2070 (hsp-16-2/GFP) worms, grown at 20°C, were treated with vehicle control (Ctrl), SQDB (100 µg/ml), GS, CIN (10 µg/ml) for 48 h starting at 2 days of age. The worms were exposed to 35°C for 2 h and transferred to 20°C for 4 h to recover before fluorescence microscopy. *Integrated Density* means the sum of the values of the pixels in the image or selection. For quantifying a population of GFP reporter animals, each 40× image was analyzed using Image J software. Data are expressed as GFP integrated pixel density obtained from at least three independent experiments with at least 10 worms in each experimental group. **Statistically significant (independent t test, P<0.01); ***statistically significant, P<0.001.

To elucidate whether the above identified lifespan extending herbs regulate a specific stress response gene, we used the transgenic *C. elegans* (CL2070) expressing GFP as a reporter for inducible *hsp-16.2* expression. Figure 2A shows representative images of *hsp-16.2*/*GFP* worms induced by a heat shock treatment for 2 hr (temperature shift from 20°C to 35°C). Upon induction by thermal stress, the GFP fluorescence is visible at the head of the worm, including the pharynx and the anterior nerve ring in the transgenic worms. The expression of *hsp-16.2* induced by heat shock was significantly enhanced in CL2070 worms fed with SQDB, GS and CIN (Fig. 2B).

**Figure 3:**
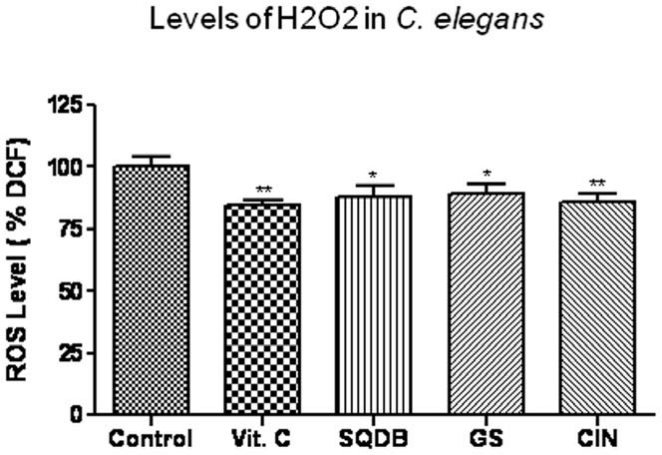
Intracellular levels of H_2_O_2_-related reactive oxygen species (ROS) in wild type *C. elegans* treated with SQDB, GS and CIN. Age-synchronized *C. elegans*, maintained and collected as described in Materials and Methods, were assayed at day 4 of age after treatment with vehicle (control), herbal formula SQDB (100 µg/ml), CIN, GS (10 µg/ml) or L-ascorbic acid (Vit.C 10 µg/ml) for 48 h. The worms were then analyzed for H_2_O_2_ levels by incubation with 50 µM DCF-DA for 2.5 h, followed by measurement of fluorescent DCF production. Results are expressed as DCF fluorescence relative to untreated controls. *Statistically significant (independent t test, P<0.05); **statistically significant (P<0.01). Results are obtained from three independent experiments with a total of 300 worms.

Oxidative free radicals have been postulated as a cause of aging and of some degenerative diseases [39]. Previously, we were able to measure H_2_O_2_-related ROS levels in *C. elegans*, and an age-dependent increase in the levels of H_2_O_2_ was observed [40]. To monitor the levels of H_2_O_2_ in wild type *C. elegans* treated with the herbs, we performed a DCF-DA fluorescence assay. We observed that in *C. elegans* treated with SQDB, GS and CIN the basal levels of H_2_O_2_ were significantly attenuated compared to untreated worms (Fig. 3). As a comparison, levels of H_2_O_2_ were also attenuated in worms fed with ascorbic acid (Vit. C). The ability of all life span-extending herbs to also enhance expression of the stress response protein *hsp-16.2* and decrease ROS levels suggests that antioxidative pathways are a nonspecific effect associated with life span extension by antioxidant herbs.

### 4. Life Span Extending Herbs Delay Amyloid-Induced Paralysis in Transgenic *C. elegans* Expressing Human Aβ

**Figure 4:**
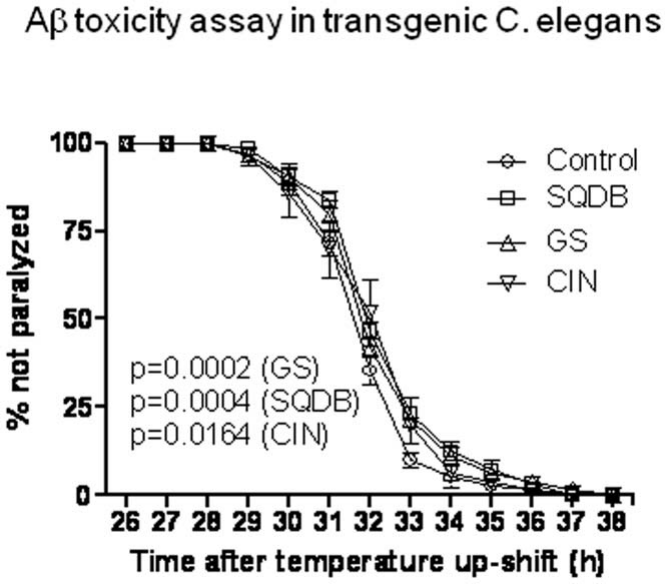
Paralysis assay in a transgenic *C. elegans* (CL4176) expressing Aβ. Age synchronized CL4176 worms were treated from egg until the L3 larval stage with CIN, GS (10 µg/ml) and SQDB (100 µg/ml). The animals were allowed to cultivate at 16°C for 38 h. At the 38 h time point, the temperature was up-shifted to 23°C. Worms began to paralyze 25 h after the temperature up-shift and were scored at 1 hr intervals. Data are expressed as percent (%) of worms not paralyzed. SQDB, GS and CIN alleviate β amyloid-induced toxicity in the transgenic *C. elegans* strain.

The *C. elegans* FOXO transcription factor, DAF-16, is repressed by activation of the DAF-2 insulin receptor and is considered a key regulator of many important biological processes including lifespan, metabolism and stress response. It has been shown that DAF-16 regulates the process that converts misfolded proteins from toxic form (oligomers) to less toxic forms (high molecular mass aggregates). Recently, it was demonstrated that decreasing expression of *daf-2* in *C. elegans*, the homolog of the mammalian IR/IGF-1R, reduces Aβ_1–42_ toxicity [41]. To determine if life span-extending herbs have an effect on protein misfolding-associated pathological behaviors, a transgenic *C. elegans* model of Aβ toxicity was used. The transgenic worms display progressive muscle paralysis after induction of transgene expression by temperature up-shift from 16°C to 23°C. The paralysis time course demonstrated (Fig. 4) that Aβ-induced muscle paralysis was significantly delayed in worms fed with SQDB (p = 0.0004), GS (p = 0.0002), and CIN (p = 0.0164).

## Discussion

In this study, we demonstrated that herbal formula SQDB and two constituent herbs *Cinnamomum cassia* bark (CIN) and *Panax ginseng* root (GS) significantly extended lifespan in *C. elegans*. The observation that herbal formula HLXL did not prolong lifespan validates the *C. elegans* model for evaluating efficacy of herbs in an organism, because the herbal formula SQDB has been used for age-related fatigue and improving wellness, whereas HLXL is more specific for anti-inflammation and used to treat arthritis.

*C. elegans* is a relevant model system for evaluating the efficacy of life span extending herbs due to the high conservation of biochemical pathways from worms to humans, and because of the similarity of many aspects of aging shared by nematodes and humans. Sarcopenia, the loss of muscle mass, occurs in both humans and C. elegans as a result of advanced age and results in corresponding behavioral declines. Intervention with ginseng has been demonstrated to slow this decline in *C. elegans* [19]. Though aging mechanisms have been well studied in model organisms such as yeast and nematodes, a feasible pharmaceutical intervention for humans has not yet been discovered. Because of the co-evolution of plants and animals, it is practical to evaluate natural compounds as potential therapies for aging and aging-related declines. Indeed, such compounds have been demonstrated to delay aging and age-related declines in model organisms [19], [20], [38], [42], [43].

Despite the common use of herbal supplements in the United States and elsewhere [2], the precise mechanisms associated with purported beneficial effects of these supplements are unknown. This lack of information is at least partially due to the fact that investigation of herbal formulas is complicated by the intricate nature of these mixtures, and an advanced method for studying individual herbs is necessary to facilitate understanding of their mechanisms. Well-characterized mutations of *C. elegans* that promote longevity, including insulin signaling and stress response, have previously been utilized to evaluate the specific mechanisms of natural compounds [38]. Similarly, multiple pathways were evaluated in this study for their involvement in lifespan-prolonging mechanistic targets of SQDB, GS, and CIN.

Oxidative stress has previously been demonstrated to be a major factor limiting lifespan in both *C. elegans* and humans [44] and as such, has gained widespread support as a preeminent theory of aging [45]. SQDB, GS, and CIN all significantly reduced levels of reactive oxygen species. However, CIN did not extend lifespan in the *mev-1* mutant (for summary see Table 6) indicating that though this herb reduces oxidative stress in the wild type worm, it likely requires an endogenous oxidative response pathway to do so. SQDB extended lifespan in the *ser-1* mutant, indicating that SQDB acts independently of serotonin signaling in prolonging lifespan. Additionally, GS extended lifespan in the *daf-16* mutant, suggesting that GS does not require insulin signaling to promote longevity. However, we observed that CIN failed to promote longevity in *ser-1* and *daf-16* mutants (Table 6), indicating that serotonin and insulin signaling are both involved in the beneficial effects exerted by CIN. Cinnamon has previously been shown to activate insulin receptor kinase activity and autophosphorylation of the insulin receptor in rats [46]. Combined with our data demonstrating that CIN requires a downstream member of the insulin signaling pathway in *C. elegans*, it is rational to conclude that CIN acts on the insulin receptor to elicit a beneficial response in the nematode. Modulation of serotonin by antidepressants has been demonstrated to enhance lifespan in C. elegans [22] and our results indicate that the serotonin pathway is required for CIN induced longevity. However, the manner in which biogenic amine neurotransmitters affect lifespan, and how CIN may modulate that mechanism requires further investigation. It is clear from our data that CIN has a pluripotent effect on lifespan in *C. elegans* with multiple targets through which it exerts this effect.

Lifespan has been linked to stress response in *C. elegans*, and in fact, long-lived worms are invariably stress resistant [44], [47]. It has been suggested that pharmaceutical interventions that enhance longevity will also have therapeutic benefits for age-related diseases such as cancer and neurodegenerative disorders [48], perhaps via activation of endogenous stress response pathways. Transgenic *C. elegans* models have been used previously to investigate the pathological mechanisms of amyloid beta (Aβ), a contributing factor to the development and progression of Alzheimer's disease (AD) [15, 49, and 50]. In this study, we investigated the effects of SQDB, CIN, and GS on Aβ-induced paralysis in a transgenic *C. elegans*. SQDB, CIN, and GS all delayed paralysis in this assay, indicating a potential beneficial effect of these herbs in an age-related disease. Additionally, we observed that these compounds significantly increased expression of heat shock protein, an endogenous stress response that has previously been demonstrated to diminish Aβ-induced pathological phenotypes in transgenic *C. elegans*. These data support the theory that longevity promoting compounds may also provide a beneficial effect in age-related diseases, and we have identified several new herbal compounds that may be further investigated as potential AD therapeutics. These compounds present exciting and rational new leads because the stress response mediated by heat shock proteins is a highly conserved physiological process in both invertebrate and vertebrate models [51].

Cinnamon is one of the most widely used herbal medicines with diverse bioactive effects. Our study demonstrated its lifespan prolonging, anti-stress and anti-proteotoxicity effects in an organism. These observations are supported by recent *in vitro* studies that demonstrated CIN to exert protection against oxidative stress [52] and mitochondrial dysfunction [53], to have anti-tumor properties [54], and to inhibit Alzheimer disease associated tau aggregation [55]. A systematic evaluation of more than 30 herb found that cinnamon is among the most potent antioxidant herbs [56] and represents an important source of dietary antioxidants [57] as well as a relevant source for candidate drugs in oxidative stress-related diseases [58]. Furthermore, studies using type II diabetic animals reported anti-diabetic effects of cinnamon extract administered at 200 mg/kg for 6 weeks [59]. Multiple human clinical trials have also provided evidence for anti-diabetic effects of cinnamon in patients and healthy subjects [60]–[66]. Mechanistic studies of several biomarkers suggest that the effect of cinnamon is mediated via regulation of multiple genes involved in insulin sensitive and lipogenesis pathways [67]–[69]. Our results provide further evidence for this view by providing genetic targets of cinnamon in an organism.

Though multiple genetic and pharmaceutical interventions have been discovered that increase lifespan in *C. elegans*, the translation of these effects to humans remains unknown. Our data and that of several others illustrate the power of *C. elegans* in screening for herb mechanisms [15], [37], [38]. Further studies that use mammalian systems to build on the discoveries made in *C. elegans* will lead to a better understanding of how these compounds apply to humans.

It should be noted that bioavailability studies can be done using *C. elegans* [70], though they were not performed in this study due to the complexity of the herbal formulas. Future studies may incorporate this type of assay to provide a better understanding of the pharmacokinetic properties of herbal compounds. It must also be noted that Zarse et al observed significant differences in lifespan between the liquid culture technique utilized in this study and solid media culture. It has been proposed that worms in liquid culture exist in a state of caloric restriction, though this conjecture has not been evaluated [71].

The present study validated a simple animal model of aging for mechanistic studies and evaluation of efficacy of herbal supplements. This model can be used for future investigation of longevity promoting herbs or compounds and concurrent mechanisms of action.

## Materials and Methods

### Preparation of HLXL, SQDB and Chemicals

The method of HLXL preparation has been previously reported [26], [27]. In brief, the 11 herbs used in the HLXL were purchased in Beijing (Tong Ren Tang pharmaceutical company, Beijing, China) and authenticated by the Beijing Institute of Material Medica following Chinese pharmacopoeia guidelines (The Pharmacopoeia Commission of PRC, 2000). They were ground into powder (∼80 mesh), and both non-polar and polar components were completely extracted with 70% aqueous acetone at room temperature. The crude extract was concentrated under reduced pressure, and the dried residue was weighed. The weight of the final HLXL extract was approximately 20∼25% that of the raw herbs. The quality of the extract was monitored by high performance liquid chromatography (HPLC) fingerprint to assure lot-to-lot consistency. The extract of each individual herb was tested for heavy metals (Hg, As, Cd) contamination before mixing.

All herbal specimens in SQDB were deposited at the Department of Herbal Pharmaceutical Development (Korea Institute of Oriental Medicine, Daejeon, Korea). Preparation of SQDB used the modified method of Korea Pharmacopoeia version 9. SQDB was composed of same amount (10 g) of each drug, a twenty-fold mass of water was added and the mixture was boiled for 2 hr at 100°C. After filtration, concentration of a filtrate by spray drying (Eyela SD1100, Japan) was carried out. The dried SQDB was maintained at 4°C before use. Mianserin, L-ascorbic acid [vitamin C (VC)] as well as other chemicals were purchased from Sigma (St. Louis, MO). Stock solutions of all chemicals were made either with 100% DMSO or with distilled water only. The final concentration of DMSO did not exceed 0.01% in the food (*Escherichia coli* strain OP50).

### Strains and Genetics

*C. elegans strains* (for a summary, see Table 2) The wild-type strain N2 (Bristol) was obtained from the *Caenorhabditis* Genetics Center (University of Minnesota, Minneapolis, MN). The following genes and alleles were used in this work: *ser-1(ok345)* (*C. elegans* Gene Knockout Consortium), *daf-16 (mgDf50)* and *mev-1(kn1) III*. The construction and characterization of the transgenic nematode strains CL4176 (*smg*-1^ts^ [*myo-3*/Aß_1–42_ long 3′-untranslated region (UTR)]) have been described previously [72], [73]. CL4176 is a temperature-sensitive mutant strain that expresses human Aβ_1–42_ only when it reaches non-permissive temperatures. The expression of Aβ in muscle cells causes paralysis in these mutants [72]. The transgenic strain *hsp-16.2*/*GFP* (CL2070) was generated and characterized by Dr. C. Link as described previously (7). CL2070 contains a jellyfish GFP reporter transgene that is under the control of the promoter for the sHSP gene *hsp-16.2*.

### *C. elegans* Maintenance and Treatment

Maintenance and manipulation of all strains of *C. elegans* have been previously described (13–18) while CL4176 was propagated at 16°C on solid nematode growth medium (NGM) seeded with 100 µl spots of *E. coli* (OP50) for food. To prepare age synchronized animals, nematodes were transferred to fresh NGM plates after reaching reproductive maturity at 3 d of age and allowed to lay eggs for 4–6 h. Isolated hatchlings from the synchronized eggs (day 1) were cultured on fresh NGM plates in either a 20°C or a 16°C (for CL4176) temperature-controlled incubator (model 2005; Sheldon Manufacturing, Cornelius, OR). The worms were fed with the herbs either from stage L1 (1 d of age) or starting from egg. The stock solutions of herbs were diluted in distilled water (DDW), or food source of *C. elegans*. For the liquid culture life span assay, 10 µL of stock solution (1.5 mg/ml formulas and single herbs) was added to the 96 well plate including stage L1 worms to obtain a final concentration of 100 µg/ml. The worms for thermal stress assay, oxidative stress assay and paralysis assay were fed with 10 µL of stock solution in the OP50 food source, seeded on NGM plates and starting from egg. Final concentrations of herbal drugs ranged from 10 µg/ml to 100 µg/ml.

### Lifespan Assay in Liquid Medium

Lifespan was assessed in liquid medium [22] (S-complete medium with 50 µg/ml carbenicillin and 0.1 µg/ml fungizone) in 96-well plates containing, respectively, 150 µL total volume, 10–15 nematodes, and 6 mg/ml freshly prepared *E. coli* OP50 per well. Age-synchronized nematodes were seeded as L1 larvae and plates were sealed with tape (Nunc) to prevent evaporation. To prevent self-fertilization, 5-fluoro-2′-deoxyuridine (0.12 mM final) (Sigma) was added 42–45 h after seeding. Worms were treated with final concentrations of 100 µg/ml of herb. Herbs were added 68 h after seeding (day 1 of adult life) unless otherwise specified. Day 1 of the lifespan assay started 68 h after seeding the animals into plates. The fraction of animals alive per well was scored microscopically on the basis of movement. Before counting, each plate was put onto a plate rotator for 1–2 min. Strong microscope light (visual or ultraviolet) effectively stimulated movement even in old animals. Using this assay, *daf-16* aging mutants showed alterations in lifespan similar to those reported using standard conditions (agar plates).

### Fluorescence Microscopy and Quantitation of hsp-16.2 Expression

Using the CL2070 strain, we used thermal stress to induce *hsp-16.2* gene expression. The worms were fed with herbal drugs (final concentration of 10 µg/ml or 100 µg/ml) starting from egg until completion of the assay. The *hsp-16.2*/*GFP* worms, synchronized and maintained as stated above, were exposed to 35°C for 2 h. Worms were then allowed to recover in their normal environment at 20°C for 4 h before pictures were taken. After induction, the expression of *hsp-16.2* was measured by directly observing the fluorescence of the reporter *GFP*. Epifluorescence images were acquired at the same exposure parameter using the 40× objective of a microscope (BX 60, Olympus, Tokyo, Japan) equipped with a digital camera (Micropublisher 5.0, QIMAGING, Burnaby, BC, Canada). For quantifying a population of GFP reporter animals, each 40× image was analyzed using Image-J software (NIH).

### Analysis of Oxidative Free Radicals

The assay was conducted as described previously [40]. Worms were cultivated on agar plates and fed 10 µL of stock solutions (final concentration of 10 µg/ml or 100 µg/ml) from egg until the worms were collected. 4-day old age synchronized worms were collected in 100 µl of 0.1% PBST. Intracellular hydrogen peroxide (H_2_O_2_)-related reactive oxygen species (ROS) were measured in *C. elegans* using 2,7-dichlorofluorescein diacetate (DCF-DA; Molecular Probes). Nonfluorescent DCF-DA is a cell-permeable dye that is readily converted to 2,7-dichlorofluoroscein (DCF) by interacting predominantly with hydrogen peroxide [40]. Age-synchronized *C. elegans* treated with or without SQDB (final concentration of 10 µg/ml or 100 µg/ml) on the day after hatching for 48 h were collected into 100 µl PBST (PBS containing 0.1% Tween 20) with 60 worms from each group and 3 groups per treatment. The worms were then subjected to timed homogenization (Pellet Pestle Motor, MG Scientific) and sonication (Branson Sonifier 250, VWR Scientific) to break up the outer cuticle. 100 µl of 50 µM DCF-DA in PBS was then added to the sonicated worms. 200 µl of each sample were transferred into a 96 well plate and placed into a fluorescent microplate reader for 2 h and 30 min, set to measure fluorescence levels at 10 min intervals. For quantification, fluorescence excitation was read at 485 nm and emission read at 528 nm. For statistical analysis, the endpoint readings upon completion of the cycle were used.

### Paralysis Assays

The strain CL4176 [49], [74] maintained at 16°C was egg-synchronized on the 35×10 mm NGM plates containing either a vehicle or drug (final concentration of 10 µg/ml or 100 µg/ml) from egg until the completion of the assay. Transgene expression was induced by upshifting the temperature from 16 to 23°C 38 hours after egg laying and lasted until the end of the paralysis assay. Paralysis was scored at 1 h intervals until the last worm became paralyzed.

### Statistical Analysis

Routine statistical analyses were performed using SPSS 12 software. Comparisons and *P* value calculations for lifespan and paralysis assay were made between treated and untreated animals of the same strain using the log-rank test (Mantel–Haenszel). We observed the death of 98.6% of the animals (excluding screen). Animals that were still alive at the end of an experiment (1.4%) were analyzed as alive up to this point with unknown time of death (censoring). Wells containing more than 19 animals were excluded from the analysis. Statistical comparison between treatments for oxidative stress and thermal stress was done with unpaired Student *t* test using Prism 4 software (GraphPad Inc., San Diego, CA).
